# Metabolic health is more strongly associated with the severity and mortality of coronavirus disease 2019 than obesity

**DOI:** 10.1186/s13690-024-01372-8

**Published:** 2024-08-23

**Authors:** Hye Yeon Koo, Jae-Ryun Lee, Jin Yong Lee, Hyejin Lee

**Affiliations:** 1 Department of Family Medicine, Seoul National University Bundang Hospital, 82, Gumi-ro 173 Beon-gil, Bundang-gu, Seongnam-si, 13620 Gyeonggi-do Republic of Korea; 2 Department of Family Medicine, Seoul National University College of Medicine, Seoul, Republic of Korea; 3 Department of Health Policy and Management, Seoul National University College of Medicine, Seoul, Republic of Korea; 4 Public Healthcare Center, Seoul National University Hospital, 101 Daehak-ro, Jongno-gu, Seoul, 03080 Republic of Korea; 5 Institute of Health Policy and Management, Seoul National University Medical Research Center, Seoul, Republic of Korea

**Keywords:** Metabolic health, Metabolic syndrome, Obesity, COVID-19, Severity, Mortality

## Abstract

**Background:**

Obesity has been suggested to be associated with the coronavirus disease 2019 (COVID-19); however, it is unclear whether obesity or metabolic abnormalities accompanied by obesity have a stronger association with COVID-19 risk.

**Methods:**

This study used the Korea Disease Control and Prevention Agency database, which includes information about the COVID-19 diagnosis and mortality dates of the entire Korean population between October 2020 and December 2021 (for diagnosis) or March 2022 (for mortality). A total of 24,310,283 adults were included and classified into four metabolic obesity phenotypes: (1) metabolically healthy and normal weight (MHNW), (2) metabolically unhealthy and normal weight (MUNW), (3) metabolically healthy and obese (MHO), and (4) metabolically unhealthy and obese (MUO). COVID-19 mortality and severity were compared according to metabolic obesity phenotypes in the total population and in each age group (20–<50 years, 50–<70 years, and ≥ 70 years). Additionally, major adverse cardiovascular events (MACE) after COVID-19 infection were compared according to metabolic obesity phenotypes.

**Results:**

A total of 3, 956, 807 participants (16.3%) were diagnosed with COVID-19 during the study period. Among them, metabolically unhealthy subjects had higher mortality rates than metabolically healthy subjects (0.81% for MUNW, 0.40% for MUO, 0.23% for MHNW, and 0.19% for MHO). The rates of severe hospitalized disease were also higher in metabolically unhealthy subjects than in healthy subjects (0.59% for MUNW, 0.55% for MUO, 0.19% for MHNW, and 0.31% for MHO). In the subgroup analyses by age, similar trends were observed in subjects aged 20–50 and 50–70 years, respectively. Additionally, the incidence of total MACE was increased in metabolically unhealthy individuals.

**Conclusions:**

The study shows that metabolic health is more strongly associated with COVID-19 mortality and severity than obesity, particularly in adults aged < 70 years.

**Supplementary Information:**

The online version contains supplementary material available at 10.1186/s13690-024-01372-8.

**Table:** 

Text box 1. Contributions to the literature
- Obesity has been suggested to be associated with COVID-19, but it was unclear whether obesity or metabolic abnormalities accompanied by obesity have a stronger association with COVID-19 outcomes.
- In this study, metabolic health was more significantly associated with COVID-19 mortality and severity than with obesity, particularly in adults aged < 70 years.
- Public health strategies that implement more intensive COVID-19 prevention and treatment for metabolically unhealthy adults, especially those under 70 years of age, are needed in the era of ongoing pandemics.

## Background

In the era of the coronavirus disease 2019 (COVID-19) pandemic, obesity and metabolic health have gained interest because of their possible associations with COVID-19 infection or severity, as suggested in previous studies [1–6]. According to a meta-analysis including 220,000 subjects, obese subjects had a 1.5–fold higher risk of severe acute respiratory syndrome coronavirus 2 (SARS-CoV-2) positivity, and COVID-19 patients with obesity had a 1.5–fold higher risk of hospitalization than non-obese patients [5]. Recent investigations have suggested that poor metabolic health might also increase the risk of severe COVID-19 [6]. Several studies have been conducted regarding the impact of obesity or metabolic health on COVID-19 following these results, and some have observed an additive effect of obesity and metabolic diseases [7]. However, few researchers have investigated whether obesity or metabolic health consequences associated with obesity more strongly affect COVID-19 prognosis.

Obesity is a risk factor for various metabolic diseases, including hypertension, type 2 diabetes mellitus, and hyperlipidemia [8]. Metabolic changes caused by abnormal fat accumulation and insulin resistance are thought to be critical links between obesity and cardiometabolic complications [9]. Considering that putative mechanisms of the obesity–COVID-19 relationship include cardiometabolic deterioration, the inflammatory role of adipocytes, and impaired vascular health [10, 11], it is possible that metabolic health is a crucial component of poor prognosis in obese COVID-19 patients.

Since not all obese individuals may have metabolic abnormalities, the concept of metabolic obesity phenotype, which classifies obesity into metabolically unhealthy obesity and healthy obesity, has been suggested [9]. Consensus on the criteria for metabolic health is still lacking and novel phenotypes have also been proposed [9, 12], but the definition based on metabolic syndrome is most commonly used [9, 13]. Although several researchers have observed that metabolic health is related to COVID-19 severity [6, 14–16], only a few studies have examined the effects of metabolic and obesity phenotypes on COVID-19 simultaneously [17, 18]. However, these studies were conducted early during the COVID-19 pandemic and included only a limited number of COVID-19 patients [17, 18]. Therefore, to clarify whether obesity or metabolic health has a stronger association with COVID-19 risk, we examined the relationship between metabolic obesity phenotypes and COVID-19 prevalence, mortality, severity, and cardiovascular complications using a large nationwide population-based dataset with an extended follow-up period. Additionally, a stratified analysis by age was performed because the infection rate and severity of COVID-19 vary with age.

## Methods

### Data source

This study used data from the Korean National Health Insurance Service (NHIS) combined with the COVID-19 database from the Korea Disease Control and Prevention Agency (KDCA). As the NHIS provides universal health coverage in Korea, nearly the entire population is included in the NHIS database [19]. The NHIS database provides information on the demographics, diagnostic records, healthcare utilization, and treatment histories of its beneficiaries. Furthermore, it includes data from a national general health screening, which is provided biennially to all NHIS beneficiaries aged over 40 years or who are employed [20]. These health screening data contain anthropometric measurements, laboratory tests, and lifestyle information. In addition, the COVID-19 database of KDCA provides information on the date of COVID-19 diagnosis and mortality of the entire population in Korea, which occurs between October 8, 2020, and December 31, 2021 (for diagnosis date) or March 31, 2022 (for mortality date).

### Study population

From the NHIS database, subjects who had participated in a national general health screening at least once during the baseline period (2016–2019) and who were aged 20 years or older at the health screening date were initially included (*n* = 28,620,098). Next, subjects with missing data on metabolic obesity phenotypes, such as body mass index (BMI), systolic blood pressure (SBP), diastolic blood pressure (DBP), fasting plasma glucose (FPG), triglyceride (TG), high-density lipoprotein (HDL), and waist circumference, were excluded (*n* = 4,309,815). In the case of subjects who underwent health screening twice, the more recent health screening data were used for analysis. Finally, 24,310,283 participants were included in a study population.

### The definition of metabolic obesity phenotypes

Obesity was defined as BMI ≥ 25 kg/m^2^, and normal weight was defined as BMI < 25 kg/m^2^, according to the Asia-Pacific criteria of the World Health Organization (WHO) [8, 21].

The definition of metabolic unhealthiness varies according to literatures and might refer to the presence of any metabolic abnormality in some studies, but we adopted the most commonly used criteria based on metabolic syndrome, in order to imply high risks of adverse health outcomes [13, 22]. Metabolically unhealthy was defined as having three or more of the following five components based on the harmonized criteria of metabolic syndrome presented by major organizations in 2009 [21, 23]: (1) FPG ≥ 100 mg/dL, (2) SBP ≥ 130 mmHg or DBP ≥ 85 mmHg, (3) TG ≥ 150 mg/dL, (4) HDL < 40 mg/dL in men or < 50 mg/dL in women; and (5) waist circumference ≥ 90 cm in men or ≥ 85 cm in women. In contrast, metabolically healthy individuals were defined as those with two or fewer of these components.

Based on these definitions, study participants were classified into four groups: (1) metabolically healthy and normal weight (MHNW), (2) metabolically unhealthy and normal weight (MUNW), (3) metabolically healthy and obese (MHO), and (4) metabolically unhealthy and obese (MUO).

### Study outcome

The primary outcome was COVID-19 mortality and severity. The secondary outcomes were COVID-19 prevalence, hospitalization, length of hospital stay, and major adverse cardiovascular events (MACE). Mortality was defined as all-cause death in COVID-19 patients within one month of medical facility usage with COVID-19 diagnosis. The severity of COVID-19 was classified into three groups (ambulatory state, hospitalized mild disease, or hospitalized severe disease), except for those who died, based on the WHO’s COVID-19 severity scale [24]. Hospitalization was defined as admission after COVID-19 diagnosis for one or more days in all patients, including those who died. The length of hospital stay was calculated in days.

MACE was analyzed to determine the cardiovascular risk after COVID-19 infection by metabolic obesity phenotypes. MACE was defined as myocardial infarction (I21-I23), stroke (I60-I64), heart failure (I11.0, I13.0, I13.2, I50), unstable angina (I20.0), cardiac death (death within one month after admission under the diagnostic code starting with I), and total MACE (composite outcome) [25] based on the International Classification of Diseases-10th Revision (ICD-10) codes. For this analysis, a cohort of subjects without prior MACE (from 2016 until the COVID-19 diagnosis date) and diagnosed with COVID-19 (*n* = 3,638,727) was constructed. The study cohort was followed from the date of COVID-19 diagnosis to the date of the MACE event, death, or March 31, 2022, whichever came first.

### Covariates

Baseline age, sex, socioeconomic status, region, Charlson Comorbidity Index (CCI), and comorbidities were extracted from the NHIS claims database. Age groups were categorized as 20–50, 50–70, and ≥ 70 years [3]. Socioeconomic status (SES) was trichotomized based on insurance premiums. The region of residence was categorized into Seoul metropolitan area and other areas (Daegu and Gyeongsangbuk Province and other areas). CCI was calculated using claims data and classified into three groups (0, 1–2, ≥ 3) [26]. Comorbidities included hypertension (I10, I15), diabetes mellitus (E10, E118, E119, E13, E149), and dyslipidemia (E78) and were confirmed using ICD–10 codes. Information about lifestyle (smoking and drinking) based on questionnaires, anthropometric measurements, and blood tests were extracted from the national health screening database.

### Statistical analysis

Subjects with missing data on metabolic-obesity phenotypes were excluded from the analysis. Descriptive statistics were used to examine the baseline characteristics of each metabolic-obesity phenotype. The COVID-19 prevalence was calculated as the number of patients per 100,000. Differences in prevalence according to metabolic obesity phenotypes in the total study population and each age–and sex–subgroup were examined. Among the subjects diagnosed with COVID-19, the COVID-19 mortality, severity, hospitalization rate, and length of hospital stay were also compared with the metabolic obesity phenotypes (in the total population and each age group). For comparisons, analysis of variance (ANOVA) was used for continuous variables, and the chi-squared or Fisher’s exact test was used for categorical variables. In addition, factors affecting the prevalence and mortality rate of COVID-19 were evaluated using multivariable logistic regression analysis with adjustment for metabolic obesity phenotypes, age, sex, region, SES, CCI, smoking, and drinking.

Cox proportional hazards analysis was performed to evaluate the associations between metabolic phenotypes and incident MACE following COVID-19 diagnosis. The proportional hazard assumption was evaluated using the Schönfeld test. Model 1 was unadjusted, Model 2 was adjusted for age and sex, and Model 3 was additionally adjusted for region, SES, CCI, smoking, and drinking.

All statistical tests were two-sided; a *P* value < 0.05 was considered statistically significant. All analyses were conducted using the SAS Enterprise Guide version 8.2.

## Results

### Baseline characteristics

The baseline characteristics of the study population are presented in Table 1. Among the study population (*n* = 24,310,283), 13,765,872 (56.6%), 1, 469, 604 (6.0%), 5, 364, 884 (22.1%), and 3, 709, 923 (15.3%) had MHNW, MUNW, MHO, and MUO, respectively. Metabolically unhealthy subjects (MUNW & MUO) were older, smoked more, had higher CCI, and had more comorbidities and prior MACE history compared with metabolically healthy subjects (MHNW & MHO) (all *p* < 0.001).

**Table 1:** Baseline^a^ characteristics of the study population in Korea according to metabolic obesity phenotypes

	Total		Metabolic obesity phenotypes								*p*-value
	(*N* = 24,310,283)		MHNW (*N* = 13,765,872)		MUNW (*N* = 1,469,604)		MHO (*N* = 5,364,884)		MUO (*N* = 3,709,923)		
**Variables**	N or Mean	% or SD	N or Mean	% or SD	N or Mean	% or SD	N or Mean	% or SD	N or Mean	% or SD	
**Sex**											< 0.0001
Male	12,160,665	50.02	5,962,576	43.31	770,807	52.45	3,127,870	58.3	2,299,412	61.98	
Female	12,149,618	49.98	7,803,296	56.69	698,797	47.55	2,237,014	41.7	1,410,511	38.02	
**Age group**,** years**											< 0.0001
20–49	10,382,476	42.71	6,419,134	46.63	276,647	18.82	2,380,449	44.37	1,306,246	35.21	
50–69	10,635,451	43.75	5,751,712	41.78	777,037	52.87	2,364,376	44.07	1,742,326	46.96	
≥ 70	3,292,356	13.54	1,595,026	11.59	415,920	28.3	620,059	11.56	661,351	17.83	
**SES**											< 0.0001
High	11,031,667	45.38	6,113,585	44.41	690,345	46.97	2,482,730	46.28	1,745,007	47.04	
Middle	7,733,076	31.81	4,470,515	32.48	426,914	29.05	1,702,018	31.73	1,133,629	30.56	
Low	5,545,540	22.81	3,181,772	23.11	352,345	23.98	1,180,136	22	831,287	22.41	
**Region**											< 0.0001
Seoul Metropolitan area	11,873,157	48.84	6,804,397	49.43	659,182	44.85	2,602,810	48.52	1,806,768	48.7	
Other	12,437,126	51.16	6,961,475	50.57	810,422	55.15	2,762,074	51.48	1,903,155	51.3	
**CCI**											< 0.0001
0	11,347,378	46.68	7,032,194	51.08	480,971	32.73	2,500,626	46.61	1,333,587	35.95	
1 ~ 2	9,156,171	37.66	5,047,583	36.67	577,780	39.32	2,064,073	38.47	1,466,735	39.54	
≥ 3	3,806,734	15.66	1,686,095	12.25	410,853	27.96	800,185	14.92	909,601	24.52	
**Hypertension**											< 0.0001
No	17,703,166	72.82	11,263,186	81.82	784,437	53.38	3,753,712	69.97	1,901,831	51.26	
Yes	6,607,117	27.18	2,502,686	18.18	685,167	46.62	1,611,172	30.03	1,808,092	48.74	
**Dyslipidemia**											< 0.0001
No	20,799,851	85.56	12,432,726	90.32	1,034,897	70.42	4,661,658	86.89	2,670,570	71.98	
Yes	3,510,432	14.44	1,333,146	9.68	434,707	29.58	703,226	13.11	1,039,353	28.02	
**Diabetes**											< 0.0001
No	16,004,170	65.83	9,972,473	72.44	734,784	50	3,432,466	63.98	1,864,447	50.26	
Yes	8,306,113	34.17	3,793,399	27.56	734,820	50	1,932,418	36.02	1,845,476	49.74	
**History of MACE (total)**											< 0.0001
No	24,304,676	99.98	13,763,784	99.98	1,469,115	99.97	5,363,485	99.97	3,708,292	99.96	
Yes	5,607	0.02	2,088	0.02	489	0.03	1,399	0.03	1,631	0.04	
**Smoking**											< 0.0001
Never	15,454,406	63.57	9,372,135	68.08	880,475	59.91	3,200,175	59.65	2,001,621	53.95	
Ex	3,753,099	15.44	1,788,761	12.99	243,096	16.54	983,943	18.34	737,299	19.87	
Current	5,102,778	20.99	2,604,976	18.92	346,033	23.55	1,180,766	22.01	971,003	26.17	
**Drinking**											< 0.0001
No	11,496,858	47.29	6,628,588	48.15	798,385	54.33	2,376,375	44.29	1,693,510	45.65	
Yes	12,813,425	52.71	7,137,284	51.85	671,219	45.67	2,988,509	55.71	2,016,413	54.35	
**BMI**,** mean (SD)**	24.14	3.76	21.92	1.96	23.10	1.51	27.13	2.96	28.47	3.77	< 0.0001
**Waist circumference (cm)**,** mean (SD)**											
** Total**	81.38	12.58	75.88	11.14	82.46	8.94	87.19	11.10	92.94	8.47	< 0.0001
** Men**	85.31	8.61	79.87	6.00	84.69	6.55	89.08	6.32	94.50	7.00	< 0.0001
** Women**	77.45	14.54	72.84	13.03	80.00	10.46	84.56	15.09	90.39	9.91	< 0.0001
**SBP (mmHg)**,** mean (SD)**	122.82	14.81	118.58	13.80	133.27	13.85	123.40	12.90	133.56	13.75	< 0.0001
**DBP (mmHg)**,** mean (SD)**	76.00	10.00	73.53	9.36	80.92	9.76	76.63	9.11	82.33	9.95	< 0.0001
**Glucose (mg/dl)**,** mean (SD)**	100.80	24.12	96.08	19.49	117.75	34.60	98.29	19.20	115.23	31.34	< 0.0001
**Total cholesterol (mg/dl)**,** mean (SD)**	195.21	39.24	193.31	37.29	196.81	45.07	197.13	39.03	198.83	43.44	< 0.0001
**Triglyceride (mg/dl)**,** mean (SD)**	124.64	72.60	101.04	54.74	195.97	79.58	120.57	61.13	189.82	84.55	< 0.0001
**HDL (mg/dl)**,** mean (SD)**	57.09	16.34	61.23	16.71	47.01	13.70	55.70	13.92	47.75	12.87	< 0.0001
**LDL (mg/dl)**,** mean (SD)**	113.30	37.36	111.93	35.70	110.85	42.47	117.41	37.13	113.42	40.92	< 0.0001
**Creatinine (mg/dl)**,** mean (SD)**	0.86	0.55	0.84	0.53	0.90	0.63	0.88	0.51	0.91	0.59	< 0.0001
**AST (IU/L)**,** mean (SD)**	26.10	21.82	24.26	20.83	28.01	25.81	27.00	20.52	30.88	24.46	< 0.0001
**ALT (IU/L)**,** mean (SD)**	25.65	25.94	21.01	21.78	27.23	27.67	29.31	27.30	36.92	32.24	< 0.0001

*MHNW* Metabolically healthy & normal weight, *MUNW *Metabolically unhealthy & normal weight, *MHO* Metabolically healthy & obese, *MUO *Metabolically unhealthy & obese, *SD *Standard deviation, *SES *Socioeconomic status, *CCI *Charlson comorbidity index, *MACE *Major adverse cardiovascular events, *BMI *Body mass index, *SBP *Systolic blood pressure, *DBP *Diastolic blood pressure, *HDL-C *High-density lipoprotein cholesterol, *LDL-C *Low-density lipoprotein cholesterol, *AST *Aspartate aminotransferase, *ALT *Alanine aminotransferase

^a^Baseline national general health screening date during 2016–2019

### The prevalence of COVID-19

Overall, 3,956,807 of 24,310,283 subjects (16.3%) were diagnosed with COVID-19 (between October 8, 2020, and December 31, 2021) (Supplementary Table S1). The prevalence rate was highest among MHO (16,843 per 100,000) and was similar in MHNW (16,495), followed by MUO (15,537), and MUNW (14,027) (*p* < 0.0001).

When sub-classified by age group, the COVID-19 prevalence rate was higher in metabolically healthy subjects (MHNW & MHO) than in unhealthy subjects (MUO & MUNW) aged 20–50 years (*p* < 0.0001). Meanwhile, in older age groups (50–70 and ≥ 70 years), obese subjects (MHO & MUO) showed a higher prevalence rate than normal-weight subjects (MHNW & MUNW) (*p* < 0.0001). Overall, no evident trend was observed in COVID-19 prevalence according to metabolic obesity phenotypes.

### The mortality, severity and hospitalization rate of COVID-19

**Fig. 1:**
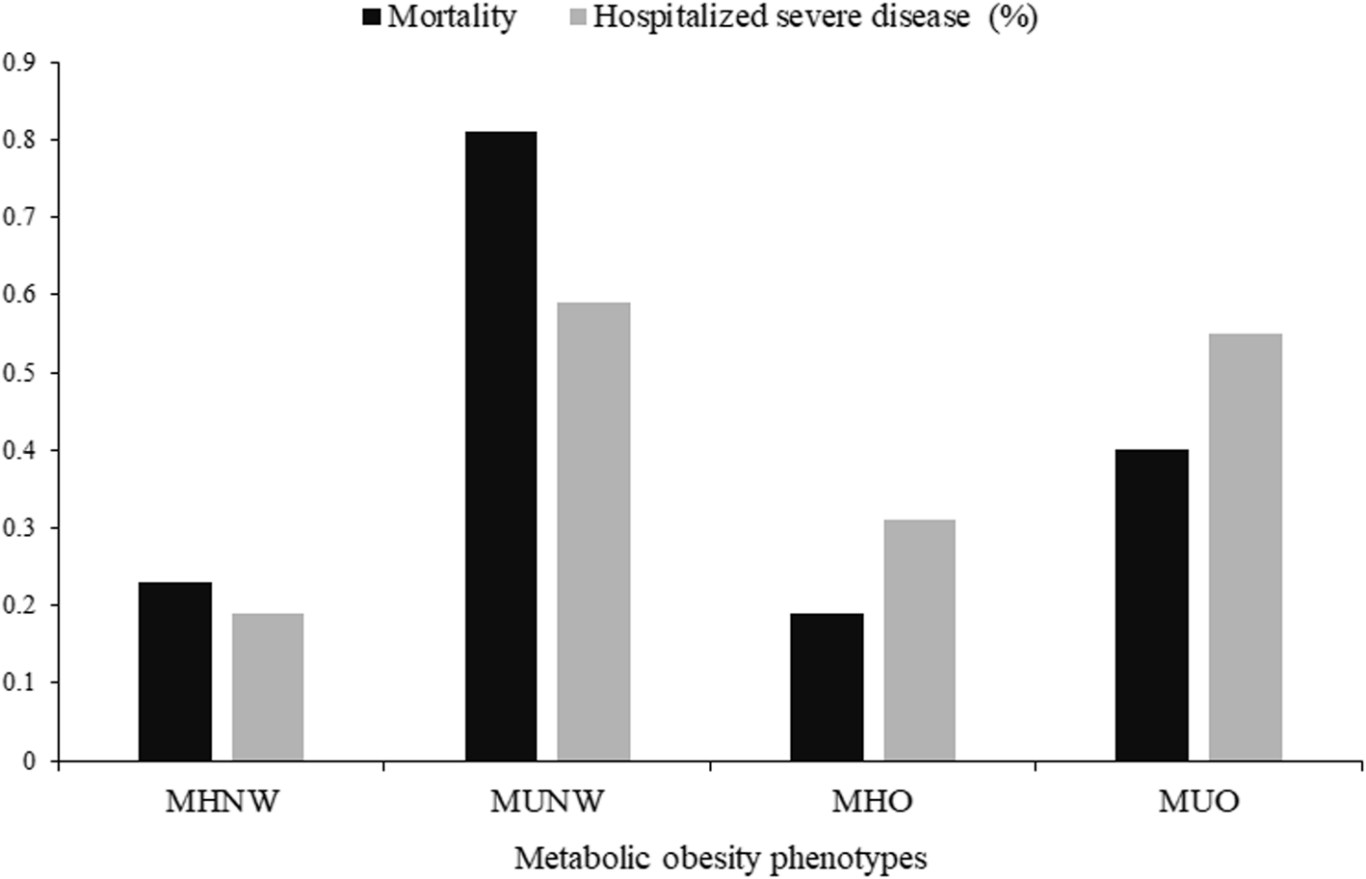
COVID-19 mortality and hospitalized severe disease rates according to metabolic obesity phenotype in Korea, October 8, 2020–March 31, 2022 COVID-19, coronavirus disease 2019; MHNW, metabolically healthy & normal weight; MUNW, metabolically unhealthy & normal weight; MHO, metabolically healthy & obese; MUO, metabolically unhealthy & obese

Table 2 presents the results of the analyses of COVID-19 mortality, severity, and hospitalization rate among COVID-19 patients (*n* = 3,956,807). The mortality, hospitalized severe disease, and total hospitalization rates were 0.27% (*n* = 10,807), 0.29% (*n* = 11,460), and 7.59% (*n* = 300,400), respectively. When comparing metabolic obesity phenotypes, metabolically unhealthy subjects showed higher mortality rates than metabolically healthy subjects, with the highest rate in MUNW (0.81%), followed by MUO (0.40%), MHNW (0.23%), and MHO (0.19%). The rates of severe hospitalized disease were also higher in metabolically unhealthy subjects than in healthy subjects (0.59% for MUNW, 0.55% for MUO, 0.19% for MHNW, and 0.31% for MHO) (Fig. 1). The total hospitalization rate also showed a similar trend to the severity rate (10.61% in MUNW, 9.04% in MUO, 6.99% in MHNW, and 7.49% in MHO). The length of hospital stay was also longer in metabolically unhealthy subjects (average 1.09 in MUNW, 0.95 in MUO, 0.70 in MHNW, and 0.78 in MHO, respectively; all in days) (all *p* < 0.0001).

**Table 2:** COVID-19 mortality, severity, hospitalization rate, and length of hospital stay according to metabolic obesity phenotype in Korea, October 8, 2020–March 31, 2022

			**Metabolic obesity phenotypes**								*p*-value
	Total COVID-19 patients (*n*=3,956,807)		MHNW (*n*=2,270,620)		MUNW (*n*=206,136)		MHO (*n*=903,631)		MUO (*n*=576,420)		
	**N**	**%**	**N**	**%**	**N**	**%**	**N**	**%**	**N**	**%**	
**COVID-19 mortality**	10,807	0.27	5,156	0.23	1,664	0.81	1,701	0.19	2,286	0.4	<0.0001
**COVID-19 Severity**											<0.0001
Ambulatory State	3,656,407	92.41	2,111,896	93.01	184,260	89.39	835,961	92.51	524,290	90.96	
Hospitalized Mild Disease	288,940	7.3	154,466	6.8	20,663	10.02	64,873	7.18	48,938	8.49	
Hospitalized Severe Disease	11,460	0.29	4,258	0.19	1,213	0.59	2,797	0.31	3,192	0.55	
**COVID-19 Hospitalization**	300,400	7.59	158,724	6.99	21,876	10.61	67,670	7.49	52,130	9.04	<0.0001
**Length of hospital stay, mean (SD)**	0.77	3.25	0.7	3.02	1.09	4	0.78	3.28	0.95	3.75	<0.0001

*COVID-19 *Coronavirus disease 2019, *MHNW *Metabolically healthy & normal weight, *MUNW *Metabolically unhealthy & normal weight, *MHO *Metabolically healthy & obese, *MUO *Metabolically unhealthy & obese, *SD *Standard deviation

Supplementary Table S2 shows the results of the analyses of COVID-19 mortality, severity, and hospitalization rates in each age group. Mortality was higher in metabolically unhealthy subjects (MUNW & MUO) than in healthy subjects (MHNW & MHO) among subjects aged 20–50 and 50–70 years. Among subjects aged ≥ 70 years, normal-weight subjects (MUNW & MHNW) showed higher mortality than obese subjects (MUO & MHO). The rates of hospitalized severe disease and hospitalization were both consistently higher in metabolically unhealthy subjects (MUNW & MUO) than in healthy subjects (MHNW & MHO) among all age groups (all *p* < 0.0001).

### Factors associated with the COVID-19 prevalence and mortality

In the multivariable logistic regression analysis, metabolic obesity phenotypes, sex, age, region, SES, CCI, smoking, and drinking were all associated with COVID-19 prevalence and mortality (Table 3). When comparing different metabolic obesity phenotypes, the adjusted odds ratio (OR) of mortality risk was highest in MUNW (OR 1.44, 95% confidence interval (CI) 1.36–1.52), followed by MUO (OR 1.06, 95% CI 1.01–1.11), and lowest in MHO (OR 0.77, 95% CI 0.73–0.82), using MHNW as the reference group.

**Table 3:** Multivariable logistic regression analyses of factors associated with COVID-19 prevalence and mortality in Korea, October 8, 2020–December 31, 2021 (for prevalence) or March 31, 2022 (for mortality)

	Prevalence (in general population)		Mortality (in COVID-19 patients)	
	Adjusted^a^ OR	95% CI	Adjusted^a^ OR	95% CI
**Metabolic obesity phenotype**				
MHNW	1 (ref.)		1 (ref.)	
MUNW	0.924	0.920–0.930	1.436	1.357–1.52
MHO	1.055	1.050–1.060	0.773	0.731–0.817
MUO	0.995	0.992–0.998	1.057	1.005–1.111
**Sex**				
Male	1 (ref.)		1 (ref.)	
Female	1.118	1.115–1.121	0.497	0.473–0.521
**Age group**				
20–49	1 (ref.)		1 (ref.)	
50–69	0.811	0.809–0.813	7.665	6.733–8.726
≥ 70	0.628	0.625–0.630	61.068	53.691–69.459
**SES**				
High	1 (ref.)		1 (ref.)	
Middle	0.936	0.934–0.939	1.123	1.071–1.177
Low	0.945	0.942–0.948	1.282	1.223–1.343
**Region**				
Seoul Metropolitan area	1 (ref.)		1 (ref.)	
Other	0.794	0.792–0.796	1.083	1.042–1.126
**CCI**				
0	1 (ref.)		1 (ref.)	
1 ~ 2	1.179	1.176–1.182	1.684	1.573–1.802
≥ 3	1.246	1.241–1.250	3.445	3.224–3.681
**Smoking**				
Never	1 (ref.)		1 (ref.)	
Ex	1.017	1.013–1.021	1.009	0.954–1.067
Current	0.582	0.580–0.584	1.632	1.528–1.744
**Drinking**				
No	1 (ref.)		1 (ref.)	
Yes	0.907	0.905–0.909	2.011	1.915–2.113

*COVID-19* Coronavirus disease 2019, *OR *Odds ratio, *CI* Confidence interval, *MHNW* Metabolically healthy & normal weight, *MUNW *Metabolically unhealthy & normal weight, *MHO *Metabolically healthy & obese, *MUO* Metabolically unhealthy & obese, *SES *Socioeconomic status, *CCI *Charlson comorbidity index

^a^Adjusted for metabolic obesity phenotypes, age, sex, SES, region, CCI, smoking, and drinking, except for the variable itself

### Incidence of MACE after the COVID-19 infection

Over an average of 1.1 months during the follow-up period, 17, 990 of 3,638,727 patients (0.49%) developed incident MACE. The incidence of total MACE was highest in the MUNW group, with a slight, insignificant increase in the MUO group (hazard ratio (HR), 95% CI: 1.31, 1.16–1.47 in MUNW, 1.09, 0.99–1.19 in MUO, and 1.03, 0.94–1.12 in MHO after total adjustment, when using MHNW as a reference) (Supplementary Table S3). A similar pattern was observed regarding the incidence of myocardial infarction, stroke, and cardiac causes of death. As for unstable angina and heart failure, the MUO group showed the highest incidence in the fully adjusted model (HR, 95% CI: 1.40, 1.18–1.65, and 1.35, 1.28–1.42, respectively).

## Discussion

Our large population-based dataset of approximately four million COVID-19 cases has shown that metabolic health is more strongly associated with mortality and severity of COVID-19 than obesity. While the prevalence of COVID-19 was higher in metabolically healthy subjects than in metabolically unhealthy subjects, COVID-19 mortality, the rate of hospitalized severe disease, and total hospitalization rate were all higher in metabolically unhealthy subjects, regardless of obesity, particularly in subjects aged < 70 years.

In our study, 5,364,888 (59.1%) of 9,070,807 obese subjects were metabolically healthy, showing that obesity does not necessarily induce significant metabolic abnormalities in all individuals, which is consistent with previous studies [9, 27]. Obesity is defined as an excessive accumulation of fat that causes chronic health risks, and it is usually diagnosed using BMI because it correlates with the body fat percentage observed in population studies [21, 28]. However, considerable evidence suggests that excess visceral fat and ectopic fat, rather than subcutaneous or total fat, are significant determinants of obesity-associated metabolic changes [29, 30]. Obese individuals with an accumulation of mostly subcutaneous fat might be at a similar cardiometabolic risk as normal-weight individuals. Therefore, metabolic health, as defined by markers of visceral or ectopic fat, such as waist circumference and TG level [29], could be a better predictor of health risks than obesity as defined by BMI. Novel findings from several small studies showing that high visceral adiposity independently associates with COVID-19 severity seem to support this hypothesis [6].

Indeed, metabolically unhealthy patients (MUO & MUNW) showed a worse outcome of COVID-19 infection, including mortality, severity, and hospitalization rate, than metabolically healthy patients (MHNW & MHO). In multivariable logistic regression, metabolic obesity phenotypes were significantly associated with COVID-19 mortality after adjustment for other relevant factors. Additionally, the ORs were higher in metabolically unhealthy subjects than in healthy subjects. Similar results were observed in a Korean retrospective cohort with a much smaller sample size than ours, including 4,069 COVID-19 cases [17]; the rate of composite critical COVID-19 outcomes increased in metabolically unhealthy individuals, with incidence rates (per 100 person-months) of 3.37 in MUO, 3.37 in MUNW, 1.64 in MHO, and 0.90 in MHNW. Furthermore, the hazard ratios of mortality were also higher in the metabolically unhealthy groups in both the unadjusted and adjusted models (2.22 in MUO, 1.90, MUNW, and 1.44 in MHO, compared with MHNW, in the adjusted model). In a study using the UK Biobank database with 3,502 hospitalized COVID-19 cases [18], the association between obesity and severe COVID-19 was attenuated but still significant after adjustment for metabolic status. However, as the definition of metabolically unhealthy status used in this study (having at least one metabolic disorder) was different from that of our study, the impact of metabolic unhealthiness might have been underestimated. Therefore, our analysis, based on a far larger sample size and a definition of metabolic unhealthiness by the presence of metabolic syndrome, has confirmed the stronger relationship between COVID-19 outcomes and metabolic health rather than obesity.

Another aspect to consider when interpreting these results is a difference in ethnic or geographical background. The prevalence of obesity in East Asia would be much lower than that in the West if Western criteria (BMI ≥ 30 kg/m^2^) is applied, but Asian populations are known to have higher visceral adiposity and diabetes risk than other populations with the same BMI range [21, 31–33]. A large number of diabetic patients in East Asia, including Korea, are observed to be in normal weight status, and it is possible that Asians with low BMI might have reduced pancreatic β-cell mass and subsequent lower insulin secretion function [31, 34]. The degree of discrepancy between obesity defined by BMI and metabolic health seems to vary across populations, and therefore associations between metabolic obesity phenotypes and COVID-19 might also differ. Future research is warranted regarding the association between metabolic health based on more rigorous definition with COVID-19 outcomes using population-based datasets from various regions.

Several explanations exist regarding the link between metabolic abnormalities and severe COVID-19 outcomes. For instance, hyperinsulinemia, insulin resistance, and subsequent hypertrophy of adipocytes can result in excessive cytokine production, chronic inflammation, and endothelial dysfunction [11, 35]. Excess visceral fat might induce the overproduction of angiotensin II and derangement of the renin-angiotensin-aldosterone system after SARS-CoV-2 infection, thereby exacerbating inflammatory responses [35, 36]. Hepatic steatosis and fibrosis can contribute to severe COVID-19 [37]; Dysregulated or increased release of hepatokines like transforming growth factor-β is suggested to associate with systematic inflammation and decreased natural killer cell function [37, 38]. Dyslipidemia may also contribute to severe outcomes because cholesterol can promote viral replication [39]. In addition, as cardiac injury and thrombosis are common in COVID-19, underlying metabolic diseases might increase the risks of cardiovascular events in combination with SARS-CoV-2 infection, resulting in poor clinical outcomes [11, 40]. Similarly, the incidence of total MACE was increased in metabolically unhealthy subjects (MUNW & MUO) in our analysis, while the difference was insignificant in the MUO group after full adjustment. Close monitoring and management of cardiovascular events might be helpful in the improvement of the COVID-19 prognosis of metabolically unhealthy patients.

The relationship between metabolic obesity phenotypes and COVID-19 mortality differed according to age in stratified analyses. While metabolic health showed a stronger association than obesity among subjects aged < 70 years, normal-weight subjects (MUNW & MHNW) showed higher mortality than obese subjects (MUHO & MHO) among subjects aged ≥ 70 years. The exact reason is unclear, but it is possible that competing mortality risks from other conditions related to lower BMI in the elderly, such as nutritional deficiency or unintentional weight loss, outweigh the risks from metabolic health [41, 42]. Similarly, in a study of BMI and COVID-19 outcomes in hospitalized patients [3], the relationship between BMI and death or mechanical ventilation was strongest in subjects aged ≤ 50 years and weakest in those aged > 70 years, and severe obesity was associated with death only in adults aged ≤ 50 years.

In our analysis, metabolically healthy subjects (MHNW & MHO) had a higher prevalence of COVID-19 than unhealthy subjects (MUO & MUNW). As metabolically healthy subjects had lower CCI and fewer comorbidities than metabolically unhealthy subjects at baseline, it is possible that these subjects were in a better general health condition and therefore were more socially active, resulting in a higher COVID-19 infection rate. Along with metabolic obesity phenotypes, sex, age, SES, region, CCI, smoking, and drinking were all associated with COVID-19 prevalence, and all these factors have also been associated with COVID-19 mortality, which is consistent with previous reports [43, 44]. The relationship between metabolic obesity phenotypes and COVID-19 mortality remained significant even after adjusting for these factors.

There were several limitations to our study. First, we could not obtain information on several inflammatory markers that may be associated with metabolic abnormalities and COVID-19 outcomes, such as cytokine levels on admission, because we used administrative data [45]. Vaccination status was not also considered, while the vaccination rate against COVID-19 was reported to be almost 90% in Korea [46]. In addition, information on metabolic health was collected from the baseline health examination results, so it might not precisely reflect the metabolic status of patients at the time of COVID-19 infection. However, most previous studies also had similar limitations [17, 18]. Also, our result might not be generalizable to other populations from different ethnic or geographic backgrounds, but we believe that our investigation provides population-level evidence on Asian ethnicity, for whom existing data is limited. The most specific strength of our study is its large population-based sample size and long follow-up period. Our large-scale data enabled us to investigate differences in associations according to age groups and to examine diverse health outcomes, including MACE. In addition, we had detailed information on anthropometric measurements and laboratory tests; therefore, we determined metabolic health following a standardized definition. Finally, our dataset also included information on various demographic and lifestyle factors not investigated in previous research.

## Conclusions

In conclusion, metabolic health was more significantly associated with COVID-19 mortality and severity than with obesity, particularly in adults aged < 70 years. Therefore, more intensive prevention and treatment strategies should be considered in metabolically unhealthy adults regarding COVID-19 in the era of ongoing pandemics.

### Supplementary Information

Supplementary Material 1.

## Data Availability

The data that support the findings of this study are available from the NHIS and KDCA. Restrictions apply to the availability of these data, which were used under the license for this study. Data are available from the authors with permission from the NHIS and the KDCA.

## Abbreviations

ANOVA: Analysis of variance
BMI: Body mass index
CCI: Charlson Comorbidity Index
DBP: Diastolic blood pressure
FPG: Fasting plasma glucose
HDL: High-density lipoprotein
HR: Hazard ratio
KDCA: Korea Disease Control and Prevention Agency
MACE: Major adverse cardiovascular events
MHNW: Metabolically healthy and normal weight
MHO: Metabolically healthy and obese
MUO: Metabolically unhealthy and obese
MUNW: Metabolically unhealthy and normal weight
NHIS: National Health Insurance Service
OR: Odds ratio
SBP: Systolic blood pressure
SES: Socioeconomic status
TG: Triglyceride

## References

[1] Bello-Chavolla OY, et al. Predicting mortality due to SARS-CoV-2: a mechanistic score relating obesity and diabetes to COVID-19 outcomes in Mexico. J Clin Endocrinol Metab. 2020;105(8):dgaa346. 32474598 10.1210/clinem/dgaa346 PMC7313944

[2] Giacomelli A, et al. 30-day mortality in patients hospitalized with COVID-19 during the first wave of the Italian epidemic: a prospective cohort study. Pharmacol Res. 2020;158:104931. 32446978 10.1016/j.phrs.2020.104931 PMC7242199

[3] Hendren NS, et al. Association of body mass index and age with morbidity and mortality in patients hospitalized with COVID-19: results from the American Heart Association COVID-19 Cardiovascular disease registry. Circulation. 2021;143(2):135–44. 33200947 10.1161/CIRCULATIONAHA.120.051936

[4] Ho FK, et al. Modifiable and non-modifiable risk factors for COVID-19, and comparison to risk factors for influenza and pneumonia: results from a UK Biobank prospective cohort study. BMJ Open. 2020;10(11):e040402. 33444201 10.1136/bmjopen-2020-040402 PMC7678347

[5] Yang J, et al. Obesity aggravates COVID-19: an updated systematic review and meta-analysis. J Med Virol. 2021;93(5):2662–74. 33200825 10.1002/jmv.26677 PMC7753795

[6] Stefan N, Birkenfeld AL, Schulze MB. Global pandemics interconnected - obesity, impaired metabolic health and COVID-19. Nat Rev Endocrinol. 2021;17(3):135–49. 33479538 10.1038/s41574-020-00462-1

[7] Stefan N, et al. Obesity and impaired metabolic health increase risk of COVID-19-related mortality in young and middle-aged adults to the level observed in older people: the LEOSS registry. Front Med (Lausanne). 2022;9:875430. 35646955 10.3389/fmed.2022.875430 PMC9131026

[8] World Health Organization. Regional Office for the western. P., The Asia-Pacific perspective: redefining obesity and its treatment. Sydney: Health Communications Australia; 2000.

[9] Tsatsoulis A, Paschou SA. Metabolically healthy obesity: criteria, epidemiology, controversies, and consequences. Curr Obes Rep. 2020;9(2):109–20. 32301039 10.1007/s13679-020-00375-0

[10] Sattar N, Valabhji J. Obesity as a risk factor for severe COVID-19: summary of the best evidence and implications for health care. Curr Obes Rep. 2021;10(3):282–9. 34374955 10.1007/s13679-021-00448-8 PMC8353061

[11] Korakas E, et al. Obesity and COVID-19: immune and metabolic derangement as a possible link to adverse clinical outcomes. Am J Physiol Endocrinol Metab. 2020;319(1):E105-9. 32459524 10.1152/ajpendo.00198.2020 PMC7322508

[12] Stefan N, Schulze MB. Metabolic health and cardiometabolic risk clusters: implications for prediction, prevention, and treatment. Lancet Diabetes Endocrinol. 2023;11(6):426–40. 37156256 10.1016/S2213-8587(23)00086-4

[13] Smith GI, Mittendorfer B, Klein S. Metabolically healthy obesity: facts and fantasies. J Clin Invest. 2019;129(10):3978–89. 31524630 10.1172/JCI129186 PMC6763224

[14] Xie J, et al. Metabolic syndrome and COVID-19 mortality among adult black patients in New Orleans. Diabetes Care. 2020;44(1):188–93. 32843337 10.2337/dc20-1714 PMC7783937

[15] Wu S, et al. Impact of metabolic syndrome on severity of COVID-19 illness. Metab Syndr Relat Disord. 2022;20(4):191–8. 34995147 10.1089/met.2021.0102

[16] Jeon WH, et al. Association of metabolic syndrome with COVID-19 in the Republic of Korea. Diabetes Metab J. 2022;46(3):427–38. 34837934 10.4093/dmj.2021.0105 PMC9171168

[17] Kim NH, et al. Metabolically unhealthy individuals, either with obesity or not, have a higher risk of critical coronavirus disease 2019 outcomes than metabolically healthy individuals without obesity. Metabolism. 2022;128:154894. 34600905 10.1016/j.metabol.2021.154894 PMC8482539

[18] Li S, et al. Metabolic healthy obesity, vitamin D status, and risk of COVID-19. Aging Dis. 2021;12(1):61–71. 33532128 10.14336/AD.2020.1108 PMC7801267

[19] Lee H, et al. Power of universal health coverage in the era of COVID-19: a nationwide observational study. Lancet Reg Health West Pac. 2021;7:100088. 33521744 10.1016/j.lanwpc.2020.100088 PMC7826087

[20] Lee H, et al. Association of cardiovascular health screening with mortality, clinical outcomes, and health care cost: a nationwide cohort study. Prev Med. 2015;70:19–25. 25445334 10.1016/j.ypmed.2014.11.007

[21] Kim BY, et al. 2020 Korean society for the study of obesity guidelines for the management of obesity in Korea. J Obes Metab Syndr. 2021;30(2):81–92. 34045368 10.7570/jomes21022 PMC8277596

[22] Eberly LE, et al. Metabolic syndrome: risk factor distribution and 18-year mortality in the multiple risk factor intervention trial. Diabetes Care. 2006;29(1):123–30. 16373907 10.2337/diacare.29.01.06.dc05-1320

[23] Alberti KG, et al. Harmonizing the metabolic syndrome: a joint interim statement of the International Diabetes Federation Task Force on Epidemiology and Prevention; National Heart, Lung, and Blood Institute; American Heart Association; World Heart Federation; International Atherosclerosis Society; and International Association for the Study of Obesity. Circulation. 2009;120(16):1640–5. 19805654 10.1161/CIRCULATIONAHA.109.192644

[24] Organization WH, Organization WH. WHO R&D blueprint novel coronavirus COVID-19 therapeutic trial synopsis. World Health Organization; 2020. pp. 1–9.

[25] Cannon CP, et al. Ezetimibe added to statin therapy after acute coronary syndromes. N Engl J Med. 2015;372(25):2387–97. 26039521 10.1056/NEJMoa1410489

[26] Lee H, et al. Comparison of complications after coronavirus disease and seasonal influenza, South Korea. Emerg Infect Dis. 2022;28(2):347–53. 35076368 10.3201/eid2802.211848 PMC8798693

[27] Kissebah AH, et al. Relation of body fat distribution to metabolic complications of obesity. J Clin Endocrinol Metab. 1982;54(2):254–60. 7033275 10.1210/jcem-54-2-254

[28] Romero-Corral A, et al. Accuracy of body mass index in diagnosing obesity in the adult general population. Int J Obes (Lond). 2008;32(6):959–66. 18283284 10.1038/ijo.2008.11 PMC2877506

[29] Després JP. Body fat distribution and risk of cardiovascular disease: an update. Circulation. 2012;126(10):1301–13. 22949540 10.1161/CIRCULATIONAHA.111.067264

[30] Liu J, et al. Impact of abdominal visceral and subcutaneous adipose tissue on cardiometabolic risk factors: the Jackson heart study. J Clin Endocrinol Metab. 2010;95(12):5419–26. 20843952 10.1210/jc.2010-1378 PMC2999970

[31] Rhee E-J. Diabetes in asians. Enm. 2015;30(3):263–9. 26435131 10.3803/EnM.2015.30.3.263 PMC4595349

[32] Mathis BJ, Tanaka K, Hiramatsu Y. Obesity vs. metabolically healthy obesity in East Asia. Encyclopedia. 2023;3(2):730–45. 10.3390/encyclopedia3020053

[33] Park Y-MM, Kim MK, Liu J. Obesity in East Asia. In: Ahima RS, editor. Metabolic Syndrome: A Comprehensive Textbook. Cham: Springer International Publishing; 2023. p. 103–21. 10.1007/978-3-031-40116-9_8.

[34] Yoon KH, et al. Selective beta-cell loss and alpha-cell expansion in patients with type 2 diabetes mellitus in Korea. J Clin Endocrinol Metab. 2003;88(5):2300–8. 12727989 10.1210/jc.2002-020735

[35] Sanoudou D, et al. Obesity, metabolic phenotypes and COVID-19. Metabolism-Clinical Experimental. 2022;128:155121. 10.1016/j.metabol.2021.155121 PMC8743503 35026232

[36] Favre G, et al. Visceral fat is associated to the severity of COVID-19. Metabolism. 2021;115:154440. 33246009 10.1016/j.metabol.2020.154440 PMC7685947

[37] Stefan N, Cusi K. A global view of the interplay between non-alcoholic fatty liver disease and diabetes. Lancet Diabetes Endocrinol. 2022;10(4):284–96. 35183303 10.1016/S2213-8587(22)00003-1

[38] Stefan N, et al. The role of hepatokines in NAFLD. Cell Metab. 2023;35(2):236–52. 36754018 10.1016/j.cmet.2023.01.006 PMC10157895

[39] Gkouskou K, et al. COVID-19 enters the expanding network of apolipoprotein E4-related pathologies. Redox Biol. 2021;41:101938. 33730676 10.1016/j.redox.2021.101938 PMC7943392

[40] Zhou F, et al. Clinical course and risk factors for mortality of adult inpatients with COVID-19 in Wuhan, China: a retrospective cohort study. Lancet. 2020;395(10229):1054–62. 32171076 10.1016/S0140-6736(20)30566-3 PMC7270627

[41] Flicker L, et al. Body mass index and survival in men and women aged 70 to 75. J Am Geriatr Soc. 2010;58(2):234–41. 20370857 10.1111/j.1532-5415.2009.02677.x

[42] Miyamoto K, et al. Evaluation of weight loss in the community-dwelling elderly with dementia as assessed by eating behavior and mental status. Asia Pac J Clin Nutr. 2011;20(1):9–13. 21393104

[43] Rashedi J, et al. Risk factors for COVID-19. Infez Med. 2020;28(4):469–74. 33257620

[44] Elliott J, et al. COVID-19 mortality in the UK Biobank cohort: revisiting and evaluating risk factors. Eur J Epidemiol. 2021;36(3):299–309. 33587202 10.1007/s10654-021-00722-y PMC7882869

[45] Li X, et al. Risk factors for severity and mortality in adult COVID-19 inpatients in Wuhan. J Allergy Clin Immunol. 2020;146(1):110–8. 32294485 10.1016/j.jaci.2020.04.006 PMC7152876

[46] Lim S, Sohn M. How to cope with emerging viral diseases: lessons from South Korea’s strategy for COVID-19, and collateral damage to cardiometabolic health. Lancet Reg Health West Pac. 2023;30:100581. 36093123 10.1016/j.lanwpc.2022.100581 PMC9442269

