# Supplementary material for: Metabolic health is more strongly associated with the severity and mortality of coronavirus disease 2019 than obesity
Source: Arch Public Health. 2024 Aug 23;82:131. doi: 10.1186/s13690-024-01372-8 (PMC11342616; doi:10.1186/s13690-024-01372-8)
Supplement: Supplementary file 1 — Supplementary Material 1. [file 13690_2024_1372_MOESM1_ESM.docx]

|  | **Metabolic obesity phenotypes** | | | | | | | | | | **p-value** |
| --- | --- | --- | --- | --- | --- | --- | --- | --- | --- | --- | --- |
|  | **Total population** | | **MHNW** | | **MUNW** | | **MHO** | | **MUO** | |  |
|  | **COVID-19 case  (N)** | **Prevalence  (N per 100,000)** | **COVID-19 case  (N)** | **Prevalence  (N per 100,000)** | **COVID-19 case  (N)** | **Prevalence  (N per 100,000)** | **COVID-19 case  (N)** | **Prevalence  (N per 100,000)** | **COVID-19 case  (N)** | **Prevalence  (N per 100,000)** |  |
| **Overall** | 3,956,807 | 16,276 | 2,270,620 | 16,495 | 206,136 | 14,027 | 903,631 | 16,843 | 576,420 | 15,537 | <0.0001 |
| **Sex** |  |  |  |  |  |  |  |  |  |  |  |
| male | 1,797,969 | 14,785 | 858,670 | 14,401 | 99,534 | 12,913 | 497,691 | 15,911 | 342,074 | 14,877 | <0.0001 |
| female | 2,158,838 | 17,769 | 1,411,950 | 18,094 | 106,602 | 15,255 | 405,940 | 18,147 | 234,346 | 16,614 | <0.0001 |
| **Age group, years** |  |  |  |  |  |  |  |  |  |  |  |
| 20-49 | 1,838,642 | 17,709 | 1,163,598 | 18,127 | 41,026 | 14,830 | 421,849 | 17,721 | 212,169 | 16,243 | <0.0001 |
| 50-69 | 1,676,584 | 15,764 | 896,826 | 15,592 | 112,379 | 14,463 | 393,175 | 16,629 | 274,204 | 15,738 | <0.0001 |
| ≥70 | 441,581 | 13,412 | 210,196 | 13,178 | 52,731 | 12,678 | 88,607 | 14,290 | 90,047 | 13,616 | <0.0001 |

**Supplementary Table S1**. COVID-19 prevalence according to metabolic obesity phenotype in Korea, October 8, 2020–December 31, 2021

COVID-19, coronavirus disease 2019; MHNW, metabolically healthy & normal weight; MUNW, metabolically unhealthy & normal weight; MHO, metabolically healthy & obese; MUO, metabolically unhealthy & obese

**Supplementary Table S2**. Subgroup analyses of mortality and severity of COVID-19 by age groups in Korea, October 8, 2020–March 31, 2022

|  |  |  | **Metabolic obesity phenotypes** | | | | | | | | **p-value** |
| --- | --- | --- | --- | --- | --- | --- | --- | --- | --- | --- | --- |
|  | **Total COVID-19 patients** | | **MHNW** | | **MUNW** | | **MHO** | | **MUO** | |  |
|  | **N** | **%** | **N** | **%** | **N** | **%** | **N** | **%** | **N** | **%** |  |
| **Age 20-49, years** |  |  |  |  |  |  |  |  |  |  |  |
| **COVID-19 mortality** | 258 | 0.01 | 130 | 0.01 | 14 | 0.03 | 55 | 0.01 | 59 | 0.03 | <0.0001 |
| **COVID-19 Severity** |  |  |  |  |  |  |  |  |  |  | <0.0001 |
| Ambulatory State | 1,735,720 | 94.4 | 1,102,132 | 94.72 | 38,432 | 93.68 | 396,869 | 94.08 | 198,287 | 93.46 |  |
| Hospitalized Mild Disease | 101,786 | 5.54 | 61,090 | 5.25 | 2,557 | 6.23 | 24,612 | 5.83 | 13,527 | 6.38 |  |
| Hospitalized Severe Disease | 1,136 | 0.06 | 376 | 0.03 | 37 | 0.09 | 368 | 0.09 | 355 | 0.17 |  |
| **COVID-19 Hospitalization** | 102,922 | 5.6 | 61,466 | 5.28 | 2,594 | 6.32 | 24,980 | 5.92 | 13,882 | 6.54 | <0.0001 |
| **Length of hospital stay, mean (SD)** | 0.54 | 2.47 | 0.5 | 2.34 | 0.61 | 2.58 | 0.58 | 2.60 | 0.65 | 2.80 | <0.0001 |
| **Age 50-69, years** |  |  |  |  |  |  |  |  |  |  |  |
| **COVID-19 mortality** | 2,652 | 0.16 | 1,094 | 0.12 | 311 | 0.28 | 574 | 0.15 | 673 | 0.25 | <0.0001 |
| **COVID-19 Severity** |  |  |  |  |  |  |  |  |  |  | <0.0001 |
| Ambulatory State | 1,547,794 | 92.32 | 832,011 | 92.77 | 102,839 | 91.51 | 362,626 | 92.23 | 250,318 | 91.29 |  |
| Hospitalized Mild Disease | 123,921 | 7.39 | 63,222 | 7.05 | 9,130 | 8.12 | 29,129 | 7.41 | 22,440 | 8.18 |  |
| Hospitalized Severe Disease | 4,869 | 0.29 | 1,593 | 0.18 | 410 | 0.36 | 1,420 | 0.36 | 1,446 | 0.53 |  |
| **COVID-19 Hospitalization** | 128,790 | 7.68 | 64,815 | 7.23 | 9,540 | 8.49 | 30,549 | 7.77 | 23,886 | 8.71 | <0.0001 |
| **Length of hospital stay, mean (SD)** | 0.80 | 3.36 | 0.74 | 3.15 | 0.88 | 3.51 | 0.83 | 3.45 | 0.94 | 3.77 | <0.0001 |
| **Age ≥70, years** |  |  |  |  |  |  |  |  |  |  |  |
| **COVID-19 mortality** | 7,897 | 1.79 | 3,932 | 1.87 | 1,339 | 2.54 | 1,072 | 1.21 | 1,554 | 1.73 | <0.0001 |
| **COVID-19 Severity** |  |  |  |  |  |  |  |  |  |  | <0.0001 |
| Ambulatory State | 372,893 | 84.44 | 177,753 | 84.57 | 42,989 | 81.53 | 76,466 | 86.3 | 75,685 | 84.05 |  |
| Hospitalized Mild Disease | 63,233 | 14.32 | 30,154 | 14.35 | 8,976 | 17.02 | 11,132 | 12.56 | 12,971 | 14.4 |  |
| Hospitalized Severe Disease | 5,455 | 1.24 | 2,289 | 1.09 | 766 | 1.45 | 1,009 | 1.14 | 1,391 | 1.54 |  |
| **COVID-19 Hospitalization** | 68,688 | 15.56 | 32,443 | 15.43 | 9,742 | 18.47 | 12,141 | 13.7 | 14,362 | 15.95 | <0.0001 |
| **Length of hospital stay, mean (SD)** | 1.63 | 5.06 | 1.59 | 4.94 | 1.91 | 5.49 | 1.47 | 4.89 | 1.72 | 5.23 | <0.0001 |

COVID-19, coronavirus disease 2019; MHNW, metabolically healthy & normal weight; MUNW, metabolically unhealthy & normal weight; MHO, metabolically healthy & obese; MUO, metabolically unhealthy & obese; SD, standard deviation

**Supplementary Table S3.** Cox proportional hazards analysis of major adverse cardiovascular events (MACE) after COVID-19 infection according to metabolic obesity phenotypes in Korea, October 8, 2020–March 31, 2022

|  | **Metabolic obesity phenotype** | **N** | **Event number** | **Duration  (person-months)** | **Incidence rate*** | **Model 1 HR (95% CI)** | **Model 2 HR (95% CI)** | **Model 3 HR (95% CI)** |
| --- | --- | --- | --- | --- | --- | --- | --- | --- |
| **Total MACE** | |  |  |  |  |  |  |  |
|  | MHNW | 2,129,629 | 8,620 | 2220598.0 | 3.9 | 1 (ref.) | 1 (ref.) | 1 (ref.) |
|  | MUNW | 176,256 | 1,705 | 191037.5 | 8.9 | 2.342 (2.224-2.467) | 1.428 (1.272-1.603) | 1.306 (1.163-1.467) |
|  | MHO | 828,659 | 3,979 | 903757.1 | 4.4 | 1.156 (1.113-1.2) | 1.043 (0.955-1.138) | 1.025 (0.939-1.12) |
|  | MUO | 504,183 | 3,686 | 566322.8 | 6.5 | 1.73 (1.664-1.798) | 1.175 (1.072-1.289) | 1.081 (0.985-1.186) |
| **Myocardial infarction** | | |  |  |  |  |  |  |
|  | MHNW | 2,129,629 | 855 | 2249430.9 | 0.4 | 1 (ref.) | 1 (ref.) | 1 (ref.) |
|  | MUNW | 176,256 | 158 | 196130.1 | 0.8 | 2.168 (1.83-2.569) | 1.461 (1.231-1.736) | 1.37 (1.153-1.628) |
|  | MHO | 828,659 | 398 | 917895.3 | 0.4 | 1.163 (1.033-1.31) | 1.091 (0.968-1.229) | 1.08 (0.959-1.218) |
|  | MUO | 504,183 | 311 | 579213.3 | 0.5 | 1.462 (1.284-1.665) | 1.19 (1.044-1.356) | 1.131 (0.992-1.29) |
| **Stroke** |  |  |  |  |  |  |  |  |
|  | MHNW | 2,129,629 | 1,608 | 2246358.7 | 0.7 | 1 (ref.) | 1 (ref.) | 1 (ref.) |
|  | MUNW | 176,256 | 358 | 195412.9 | 1.8 | 2.585 (2.305-2.898) | 1.428 (1.272-1.603) | 1.306 (1.163-1.467) |
|  | MHO | 828,659 | 721 | 916845.2 | 0.8 | 1.109 (1.015-1.21) | 1.043 (0.955-1.138) | 1.025 (0.939-1.12) |
|  | MUO | 504,183 | 636 | 577808.3 | 1.1 | 1.561 (1.424-1.712) | 1.175 (1.072-1.289) | 1.081 (0.985-1.186) |
| **Cardiac cause of death** | | | |  |  |  |  |  |
|  | MHNW | 2,129,629 | 1,349 | 2252811.6 | 0.6 | 1 (ref.) | 1 (ref.) | 1 (ref.) |
|  | MUNW | 176,256 | 415 | 196745.6 | 2.1 | 3.634 (3.255-4.056) | 1.629 (1.458-1.819) | 1.455 (1.302-1.625) |
|  | MHO | 828,659 | 526 | 919666.6 | 0.6 | 0.974 (0.881-1.078) | 0.919 (0.831-1.017) | 0.915 (0.828-1.013) |
|  | MUO | 504,183 | 701 | 580495.0 | 1.2 | 2.093 (1.91-2.293) | 1.422 (1.298-1.558) | 1.294 (1.181-1.419) |
| **Unstable angina** | |  |  |  |  |  |  |  |
|  | MHNW | 2,129,629 | 421 | 2251080.6 | 0.2 | 1 (ref.) | 1 (ref.) | 1 (ref.) |
|  | MUNW | 176,256 | 65 | 196510.7 | 0.3 | 1.781 (1.372-2.313) | 1.116 (0.858-1.451) | 1.021 (0.784-1.328) |
|  | MHO | 828,659 | 234 | 918581.8 | 0.3 | 1.368 (1.166-1.605) | 1.245 (1.06-1.461) | 1.221 (1.04-1.434) |
|  | MUO | 504,183 | 213 | 579503.6 | 0.4 | 1.981 (1.68-2.336) | 1.521 (1.288-1.795) | 1.397 (1.182-1.651) |
| **Heart failure** | |  |  |  |  |  |  |  |
|  | MHNW | 2,129,629 | 5,131 | 2230336.5 | 2.3 | 1 (ref.) | 1 (ref.) | 1 (ref.) |
|  | MUNW | 176,256 | 903 | 192784.2 | 4.7 | 2.082 (1.94-2.235) | 1.378 (1.283-1.481) | 1.283 (1.194-1.378) |
|  | MHO | 828,659 | 2,440 | 908423.1 | 2.7 | 1.193 (1.137-1.252) | 1.15 (1.096-1.207) | 1.128 (1.075-1.184) |
|  | MUO | 504,183 | 2,188 | 570323.7 | 3.8 | 1.73 (1.646-1.819) | 1.44 (1.368-1.514) | 1.348 (1.281-1.419) |

*per 1,000 person-months

Model 1 : unadjusted; Model 2 : adjusted for age and sex; Model 3 : adjusted for age, sex, residence, SES, smoking, drinking, CCI

COVID-19, coronavirus disease 2019; HR, hazard ratio; CI, confidence interval; MHNW, metabolically healthy & normal weight; MUNW, metabolically unhealthy & normal weight; MHO, metabolically healthy & obese; MUO, metabolically unhealthy & obese
